# Response of soil microbial communities and rice yield to nitrogen reduction with green manure application in karst paddy areas

**DOI:** 10.3389/fmicb.2022.1070876

**Published:** 2023-01-09

**Authors:** Junyu Pu, Zhongyi Li, Hongqin Tang, Guopeng Zhou, Caihui Wei, Wenbin Dong, Zhenjiang Jin, Tieguang He

**Affiliations:** ^1^Agricultural Resource and Environment Research Institute, Guangxi Academy of Agricultural Sciences/Guangxi Key Laboratory of Arable Land Conservation, Nanning, Guangxi, China; ^2^The Guangxi Key Laboratory of Theory and Technology for Environmental Pollution Control, College of Environmental Science and Engineering, Guilin University of Technology, Guilin, Guangxi, China; ^3^Institute of Agricultural Resources and Regional Planning, Chinese Academy of Agricultural Sciences, Beijing, China

**Keywords:** green manure, karst area, soil microorganisms, dominant taxa, random forest model, rice yield

## Abstract

Fertilizer application practices are one of the major challenges facing agroecology. The agrobenefits of combined application of green manure and chemical fertilizers, and the potential of green manure to replace chemical fertilizers are now well documented. However, little is known about the impact of fertilization practices on microbial communities and tice yield. In this study, the diversity of bacterial and fungal communities, symbiotic networks and their relationship with soil function were analyzed in five fertilization treatments (N: 100% nitrogen fertilizer alone; M: green manure alone; MN_60_: green manure couple with 60% nitrogen fertilizer, MN_80_: green manure couple with 80% nitrogen fertilizer; and MN_100_: green manure couple with 100% nitrogen fertilizer). First, early rice yield was significantly higher by 12.6% in MN_100_ treatment in 2021 compared with N. Secondly, soil bacterial diversity showed an increasing trend with increasing N fertilizer application after green manure input, however, the opposite was true for fungal diversity. Microbial interaction analysis showed that different fertilizer applications changed soil microbial network complexity and fertilizer-induced changes in soil microbial interactions were closely related to soil environmental changes. Random forest models further predicted the importance of soil environment, microorganisms and rice yield. Overall, nitrogen fertilizer green manure altered rice yield due to its effects on soil environment and microbial communities. In the case of combined green manure and N fertilizer application, bacteria and fungi showed different responses to fertilization method, and the full amount of N fertilizer in combination with green manure reduced the complexity of soil microbial network. In contrast, for more ecologically sensitive karst areas, we recommend fertilization practices with reduced N by 20–40% for rice production.

**Graphical Abstract:**
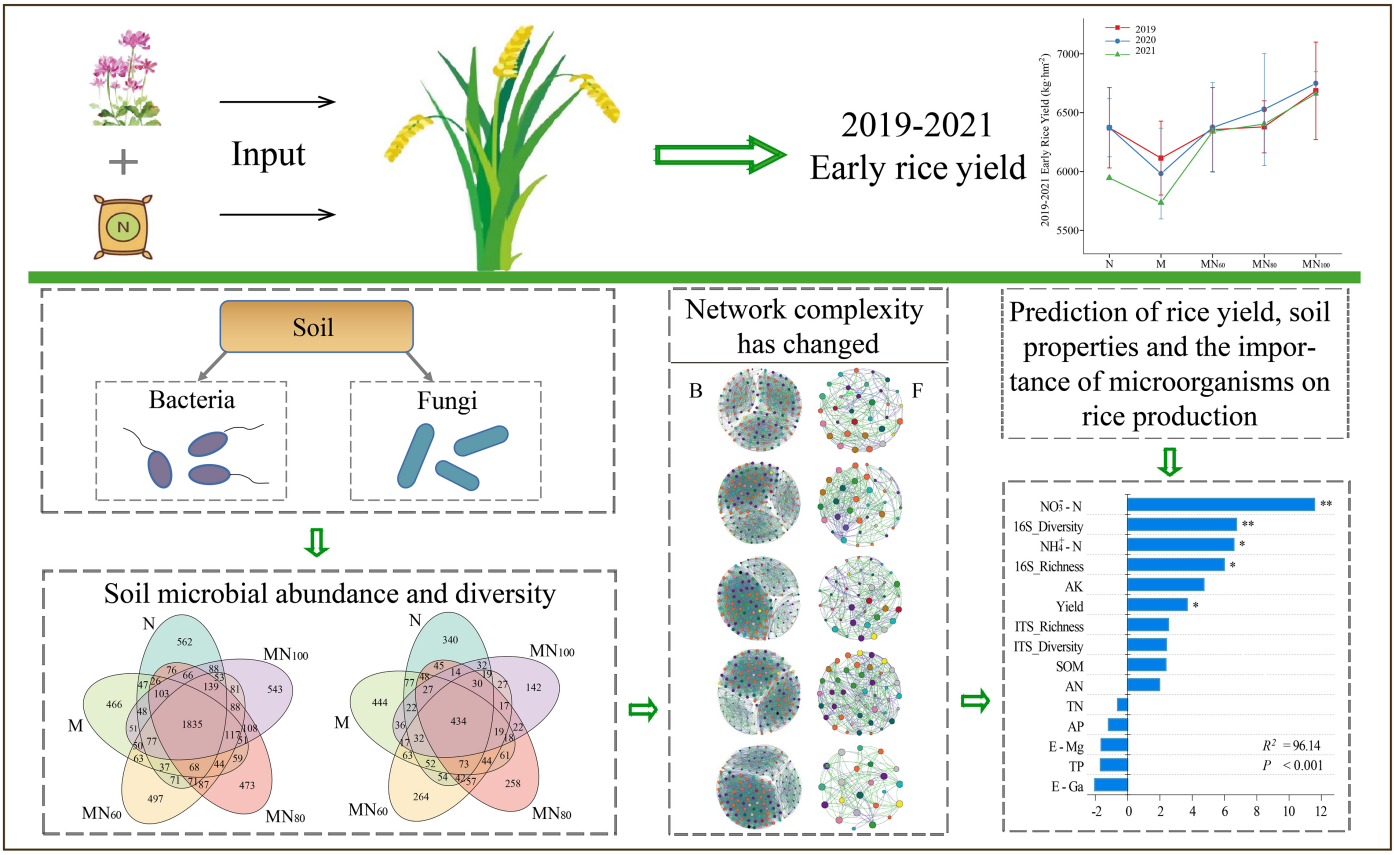

## Introduction

China is a major rice producer, accounting for about 30% of the world’s total rice production (Chen et al., 2020). Fertilizers play an important role in maintaining crop yields and are one of the most rapid and effective ways to increase crop yields. However, long-term application of large amounts of single fertilizer can threaten soil quality, which ultimately feeds into crop yield stability (Chen et al., 2011; Liu et al., 2013). Therefore, improving the agro-ecological environment and promoting sustainable crop growth has become a hot research topic.

Green manure is a completely biological nutrient that has gradually become an important alternative fertilizer for zero-growth chemical fertilizer program, which is of great significance to agricultural development (Bao et al., 2018; Zhang et al., 2023). In rice areas of southern China, the scattering of green manure crops such as Chinese milk vetch (*Astragalus sinicus* L.) in winter can make reasonably use of light and heat resources and provide a large number of organic nutrients for subsequent rice crop, and its role in reducing fertilizer and enhancing efficiency has been widely recognized (Gao et al., 2020). Previous experiments have shown that green manure is important for increasing yields, saving fertilizer, enriching soil, and improving soil nutrient absorption (Li et al., 2019; Yang et al., 2019; Zhou et al., 2020).

Soil bacteria and fungi are important components of agro-ecosystems and drivers of soil nutrient cycling and sensitive indicators for assessing soil environment (van der Heijden et al., 2008). The type and effectiveness of soil matrix is an important factor influencing microbial community changes including microbial community abundance, diversity and their functions (Sofo et al., 2019). Previous research has shown that climatic conditions (Pan et al., 2016), crop types (Guo et al., 2015) and fertilization regimes (Zhang X. et al., 2017) could interact with soil nutrients to influence microbial composition and function. Long-term input of mineral fertilizers may lead to a decrease in soil microbial diversity and abundance, resulting in decrease in soil fertility (Gao et al., 2021). Green manure planting in winter can promote crop growth and improve nutrient utilization by changing soil nutrient patterns, thus affecting microbial community’s composition and structure (Xie et al., 2017). Studies have shown that applying green manure with appropriate amounts of chemical fertilizers facilitated growth and reproduction of soil microorganisms (Zou et al., 2013; Gao et al., 2015), significantly increasing the numbers of bacteria, fungi and nitrogen-fixing bacteria (Wan et al., 2013; Gao et al., 2021). Moreover, green manure application altered community structures of soil root endophytes by increasing their richness and diversity (Zhang et al., 2013) and the number of beneficial bacteria which then promotes nutrient uptake by crop roots (Zhang X. et al., 2017) to effectively increase crop yields.

Soil microbial communities are often interconnected and form complex ecological relationships, such as parasitism, mutualism, neutrality, predation and competition, which determine their ability to maintain stability or recover from disturbances (Yang et al., 2023). Therefore, an increasing number of studies are using network analysis to understand the potential interactions within a given ecosystem. That is, species associations among microbial communities elucidate the complexity and stability of communities (Deng et al., 2012; Yuan et al., 2021). It is generally accepted that microecologies with more complex networks tend to have more active metabolic processes and faster growth rates, and their community performance is higher (Jordan, 2009; Yuan et al., 2021). Therefore, we used network analysis to better understand the effects of fertilization practices on microbial communities. Previous studies have found that changes in soil environment affect microbial network complexity due to changes in land use practices (Yang et al., 2022) and soil nitrogen accumulation reducing microbial network complexity (Ma et al., 2022), etc. However, further studies on changes in soil microbial network complexity caused by fertilization practices are needed.

As a product of carbonate rock dissolution and weathering, karst soil represents an alkaline and calcium-rich soil. The soil formation rate is slow and the thickness of soil layer is thin (Yuan, 2001; Li, 2022). Studies have shown that soil microorganisms are more abundant in karst areas than in non-karst areas with similar land use, and there are differences in microbial species interactions (Gao et al., 2012). Compared with red soils at same latitude, brownish-yellow karst soils are reported to have higher water stability and erosion resistance (Hu et al., 2013). However, relatively few studies have been conducted on the effects of fertilization practices on soil microbial community changes in karst paddy fields. Based on this, we propose the scientific conjecture that fertilizer application in karst paddy ecosystems would alter soil microenvironment by affecting microbial community structure and microbial intra- and/or inter-group interactions, which would change soil ecology and benefit rice production.

In this study, we analyzed and verified green manure-regulating effects of moderate N fertilization on environmental factors and microorganisms to understand interrelationships between environment, subsurface microbial ecology, and aboveground crop productivity. A three-year field experiment was conducted on a typical brownish-yellow rice soil in karst region to elucidate how fertilization affects microbial communities and soil properties, and how microbial communities feedback to crop productivity, and to verify the optimal fertilization strategy for rice production in karst regions by comparing different N fertilizer dosages. The main objectives were to answer the following three questions: (1) What will be the effect of N fertilizer reduction with green manure on rice production, (2) How does the reduction of nitrogen fertilizer with green manure affect the diversity, community structure and network complexity of bacteria and fungi, and (3) What is the importance of soil microbial diversity and community in maintaining productivity and soil environment of karst rice.

## Materials and methods

### Site description

The field experiment was conducted in Dingdian village (23°0′41″N, 107°51′21″E), Natong Town, Guangxi Province, China. The area has a subtropical monsoon climate with annual average temperature of 21.6°C, precipitation of about 1,300 mm and average altitude of 64 m. The soil is brownish-yellow rice soil from karst area. The basic physical properties of soil organic matter (SOM), total N (TN), available N (AN), available P (AP), available K (AK) and pH are 31.3 g/kg, 1.90 g/kg, 136 mg/kg, 19.4 mg/kg, 161 mg/kg, and 6.81, respectively.

### Experimental design and crop management

Five treatments were established in the experiments: (i) 100% nitrogen fertilizer alone (N), (ii) green manure alone (M), (iii) green manure couple with 60% nitrogen fertilizer (MN_60_), (iv) green manure couple with 80% nitrogen fertilizer (MN_80_), and (v) green manure couple with 100% nitrogen fertilizer (MN_100_). We used the treatments of N, M and MN_100_ previous determined in Pu et al. (2022). Each treatment was performed in three replications and arranged in a randomized block design. Each experimental plot area was 20.7 m^2^ and separated by a ridge to prevent the movement of water and nutrients between plots.

The positioning test began in October 2018 and samples were collected for test analysis in middle of July 2021. A double-cropping system for rice was adopted. The green manure variety was Chinese milk vetch (*Astragalus sinicus* L.), sowed evenly 1–2 weeks before the late rice harvest with a seeding rate of 30 kg/hm^2^, and the full amount was returned to field *in situ* at the full flowering stage (March 22, 2021). The test rice (Guiyu 9) was transplanted on April 7 and harvested on July 13, 2021. The paddy field was flooded and exposed to sun for 1 week at tillering stage without irrigation of 2 weeks before harvest. The fertilizer types were urea (containing 46.4% Nitrogen), calcium superphosphate (containing 18.0% P_2_O_5_), and potassium chloride (containing 60% K_2_O), respectively. The 100% N fertilizer treatment was applied with 180 kg/hm^2^ of urea, 90 kg/hm^2^ of phosphorus and 120 kg/hm^2^ of potassium. Potassium fertilizer and phosphorus fertilizer were applied once to paddy fields, while nitrogen fertilizer was applied several times according to the ratio base: tillering: tapping = 4:3:3.

### Rice and soil sampling

The field experiment was conducted for 3 consecutive years. Rice yield and associated factors were measured at the harvest stage of early rice. Soil samples were collected from surface soil (0–20 cm) of each plot on July 13, 2021. Five soil cores were randomly sampled from each plot and homogenized to reduce variability. Each fresh soil sample was divided into two sub-samples. One part was used for analysis of soil microorganism and the other was air-dried and passed 10/100 mesh sieve for analysis of soil chemical properties.

### Chemical analysis

Soil pH was tested with a soil-to-water ratio of 1:2.5 (*m*:*v*). Soil organic matter (SOM) was determined using potassium dichromate oxidation method. Soil total nitrogen (TN) was determined with Kjeldahl method, alkaliolytic nitrogen (AN) was determined by alkaliolytic nitrogen diffusion method with ferrous sulfate reductant, and filtrate concentrations of ammonium nitrogen (NH_4_^+^–N) and nitrate nitrogen (NO_3_^−^–N) were analyzed with a discrete auto-analyzer (SmartChem TM200, United States). Exchangeable calcium and magnesium were determined using ammonium acetate exchange-atomic absorption spectrophotometer method. Available phosphorus (AP) and available potassium (AK) were measured as previously described by Lu (2000). Available N was measured by diffusion method with ferrous sulfate reductant. Available phosphorus was determined by molybdenum-antimony counterstain method with sodium bicarbonate extraction, and available potassium was determined by ammonium acetate exchange flame photometry described by Lu (2000). Some data of pH, SOM, AP, AK, AN, TN, E-Ga and E-Mg were from our previoustreatments of N, M and MN100 (Pu et al., 2022).

### Bioinformatics analysis

Soil microbial sequencing was conducted by Guangdong Meige Gene Technology Co., Ltd. on the Illumina NovaSeq high-throughput sequencing platform. The primer sequences of 515F (GTGCCAGCMGCCGCGGTAA) and 907R (CCGTCAATTCMTTTRAGTTT) were selected to amplify the V4–V5 segment of bacterial 16S rRNA gene, and the PCR amplification conditions were: denaturation 95°C, 10 s, annealing 55°C, 30 s, extension 72°C, 45 s. The cycle of denaturation–annealing–extension repeated 45 times. The primer sequences of ITS1F (TCCGTAGGTGAACCTGCGG) and ITS2-2034R (GCTGCGTTCTTCATCGATGC) were selected to amplify the ITS1-4 segment of fungal ITS gene, and the PCR amplification conditions were: denaturation 95°C, 10 s, annealing 50°C, 30 s, extension 72°C, 45 s. The cycle of denaturation-annealing-extension repeated 45 times. The sequencing results were spliced using FLASH software, and low-quality sequences were removed using the Usearch software to draw a flat with the lowest sample quality sequences. By using cluster command, a taxonomy analysis of operational taxonomic unit (OTU) representative of sequences with a 97% similarity level was performed. After randomly sampling OTU table in same sequence depth, α diversity index and β diversity distance matrix were calculated using QIIME software (Cock et al., 2010). Ultimately, a total of 9,498 bacterial OTUs (belonging to 57 phyla, 128 classes, 238 orders, 440 families and 1,204 genera), and 4,505 fungal OTUs (belonging to 8 phyla, 34 classes, 99 orders, 236 families and 548 genera) were obtained from the karst rice soil All the sequence data of the present study have been deposited in the NCBI Squence Read Archive (SRA) database under accession numbers SRR21891129-SRR21891143 (Bacteria) and SRR21901595-SRR21901609 (Fungi), and we used the treatments of N, M and MN100 previous listed in Pu et al. (2022).

### Statistical analysis

Data were subjected for analysis of variance (ANOVA) according to the experimental design (Randomize Design). Significant differences in rice yield, soil properties and relative microbial abundance among each fertilization treatment were tested by one-way ANOVA followed by Fisher LSD (*p* < 0.05) test (Buyer and Sasser, 2012). The unweighted UniFrac distance for phylogenetic relationship and Bray-Curtis for microbial communities were calculated, and principal coordinate analysis (PCoA) was performed to determine the differences in microbial taxa among the samples based on the dissimilarity (R vegan package; Zhong et al., 2020). The intra- and inter-group interactions of microbial groups were investigated by network analysis to reveal OTUs in soil microbial communities interacted using the positive or negative spearman correlations under different fertilization treatments. Positive correlations indicated mutually beneficial interactions among microorganisms, while negative correlations indicated competitive relationships. OTUs of bacteria and fungi with relative abundance ≥ 0.1% were defined as the dominant groups (Zhong et al., 2021; Yang et al., 2023), which were visualized using stacking histogram. *p*-values for co-occurrence networks were obtained by Gephi based on R “psych” package (R Studio Version 4.1.2; Qiu et al., 2021). Redundancy analysis (RDA) was conducted to determine correlations between dominant OTUs and soil factors, and the effects of environmental factors and microorganisms on rice yield were assessed using the Mantel test (Sunagawa et al., 2015). A random forest model was used to predict the indicative nature of important variables on crop yield with R “randomForest” package (Liaw and Wiener, 2002), and univariate linear regression models were performed to analyze the correlations between important variables and rice yield.

## Results

### Early rice yield and soil properties

**Figure 1:**
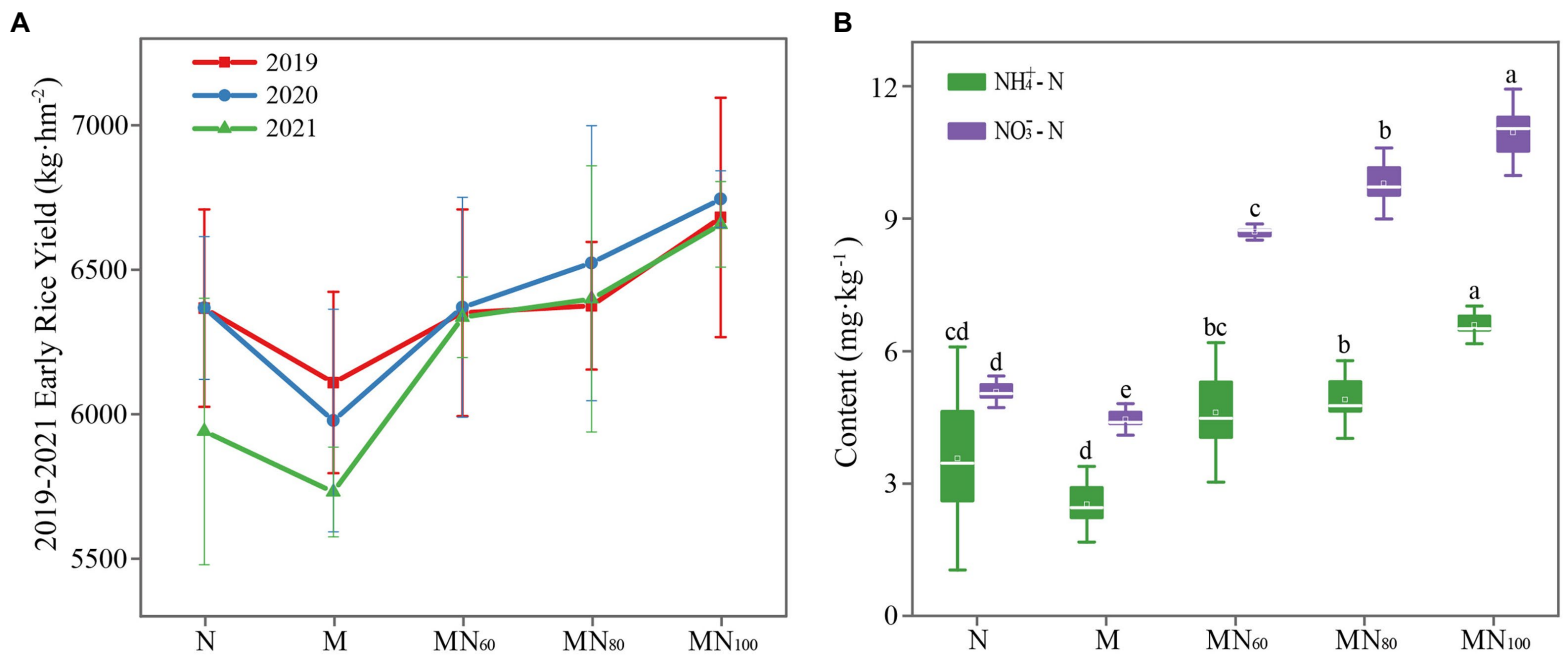
**(A)** Early rice yield of different fertilization treatments in 2019-2021. **(B)** Soil NH_4_^+^–N and NO_3_^−^–N contents under different treatments. NH_4_^+^–N: ammonium nitrogen; NO_3_^−^–N: nitrate nitrogen. The value is mean ± standard error, and different letters indicate significant difference between treatments (*p* < 0.05).

The results of early rice yield in 2019–2021 are shown in Figure 1A. In this case, there was no significant difference of early rice yield in 2019–2020 (*p* > 0.05) under different fertilizer treatments. In 2021, MN_100_ significantly increased the yield by 12.06% over N and 16.15% over M. In addition, the rice yields of MN_60_ and MN_80_ treatments showed an increasing trend comparing with N fertilizer treatments and the increases were 6.64 and 7.72%, respectively (Figure 1A).

One-way ANOVA analysis showed that the contents of SOM, AK, AN, NH_4_^+^–N and NO_3_^−^–N in paddy soils varied significantly under different fertilization treatments (Figure 1B). Among them, soil ammonium and nitrate N contents increased in MN_60_, MN_80_ and MN_100_ treatments compared with N and M. The highest ammonium and nitrate N contents were achieved in the MN_100_ treatment. The differences in soil AK content were significant at different treatments. Compared with N and M, AK content decreased significantly under MN_60_ and MN_80_ treatments, while AK content increased significantly under MN_100_ treatment. In addition, soil TN, TP, AP, E-Ca, and E-Mg did not significantly differ under different fertilization treatments (Supplementary Table 1).

### Microbial community characteristics under different fertilization treatments

#### Effects of fertilization on microbial communities

**Figure 2:**
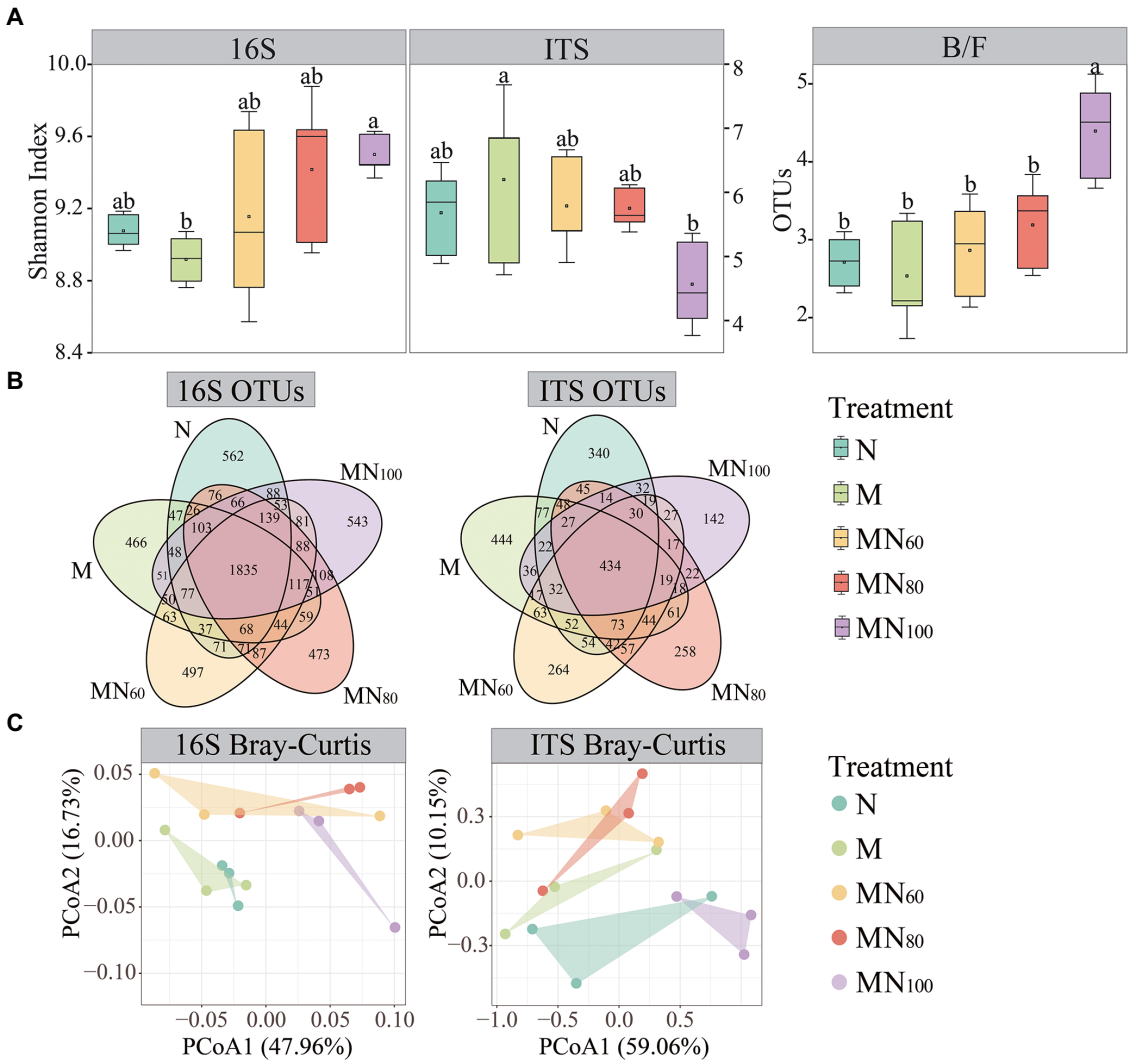
Soil microbial communities under different fertilizer treatments. **(A)** Shannon index of soil bacterial and fungal species as influenced by fertilizer treatments. Significant effects were obtained by one-way ANOVA test. Different lowercase letters indicate significant differences of bacteria and fungi among treatments, respectively (*p* < 0.05). **(B)** Venn diagram of bacterial and fungal richness in different fertilization treatments. **(C)** Principal coordinate analysis (PCoA) plot of microbial community structures based on Bray–Curtis between samples of different fertilization treatments.

Figure 2 demonstrates the opposite trend of soil bacterial and fungal Shannon indices under different fertilization treatments. The highest bacterial diversity index was observed with MN_100_ treatment and the lowest value was observed with M treatment. The opposite was the fungal diversity index compared with N treatment. The soil B/F (bacteria/fungi) ratio was significantly higher at the MN_100_ treatment than those at other treatments (Figure 2A). In addition, the Venn diagram results showed the differences in OTU categories, and numbers of 16S and ITS under different fertilization treatments (Figure 2B).

To understand the effects of fertilization measures on soil microbial community structure, PCoA based on Bray–Curtis (Beta diversity) was performed (Figure 2C). The PCoA result showed that the first two axes for bacteria explained was 64.69% and fungi was 69.21%. In addition, the result of Adonis analysis showed significant difference in bacterial (*r* = 0.298, *p* = 0.037) and fungal (*r* = 0.249, *p* = 0.044) community structure among fertilization treatments.

### Microbial community composition

**Figure 3:**
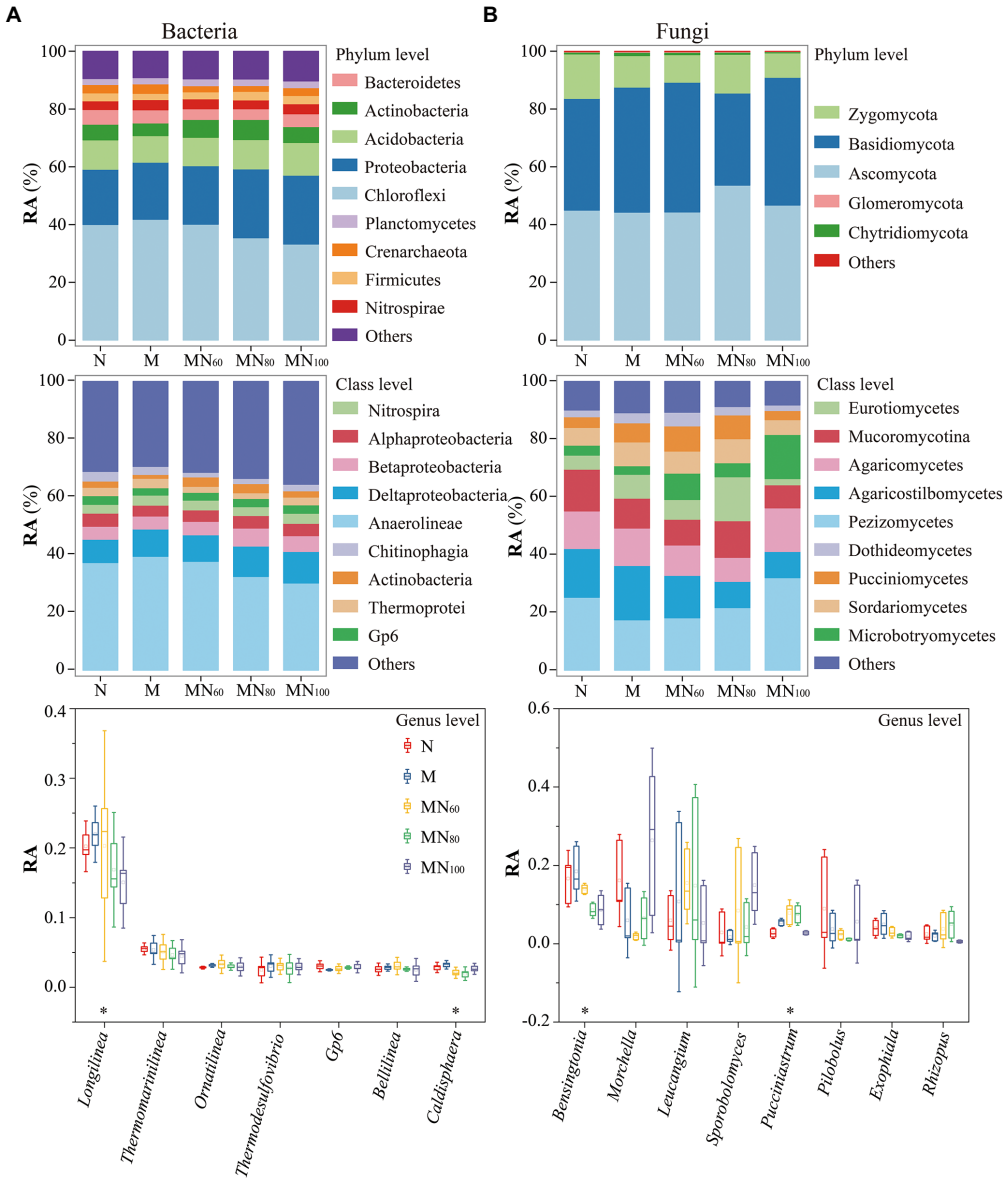
Soil microbial communities under different fertilization measures. **A,B** show the composition and abundance of soil bacteria at the taxonomic levels of phylum, order and genus, respectively. RA: Relative abundance. ^*^p < 0.05 indicates that the relative abundance of taxa at the genus level differed significantly under different fertilization treatments.

The relative abundance of major bacteria and fungi differed slightly between fertilization treatments (Figure 3). Major bacterial phyla were Chloroflexi (27.15–47.99%), Proteobacteria (17.40–25.70%), and Acidobacteria (8.44–12.20%). The main fungal species were Ascomycota (44.27–53.62%), Basidiomycota (31.87–44.90%), and Zygomycota (8.46–15.35%). In addition, MN_80_ and MN_100_ significantly increased the relative abundance of Proteobacteria by 24.83 and 24.40%, respectively, compared with N, while the relative abundance of Crenarchaeota was decreased. At class level (Figure 3B), Anaerolineae was the most abundant taxon based on 16S rRNA genes with relative abundance of 30.18–39.40%, while the relative abundance of Deltaproteobacteria was 8.09–10.92%. According to ITS genes, Pezizomycetes (17.44–32.04%) had the greatest relative abundance at phylum level.

At the genus level (Figure 3C), the relative abundance of the major bacteriophage genus *Longilinea* decreased at the MN_80_ and MN_100_ treatments compared with the N treatment, and were decreased by 16.63% (MN_80_) and 25.64% (MN_100_), respectively. The relative abundance of *Caldisphaera* also differed significantly among treatments. Compared with N, the relative abundance of *Caldisphaera* were decreased by 27.24, 32.07 and 6.90% for MN_60_, MN_80_ and MN_100_ treatments, respectively. For the major fungal genera, green manure with N fertilizer reduced the relative abundance of *Bensingtonia* and the reduction of *Bensingtonia* was 15.69, 48.62 and 48.04% for MN_60_, MN_80_ and MN_100_ treatments, respectively, compared with N. In contrast, the relative abundance of *Pucciniastrum* increased under MN_60_ and MN_80_ treatments compared with N and the increases were 65.60 and 64.54%, respectively.

### Co-occurrence network of microbial communities

**Figure 4:**
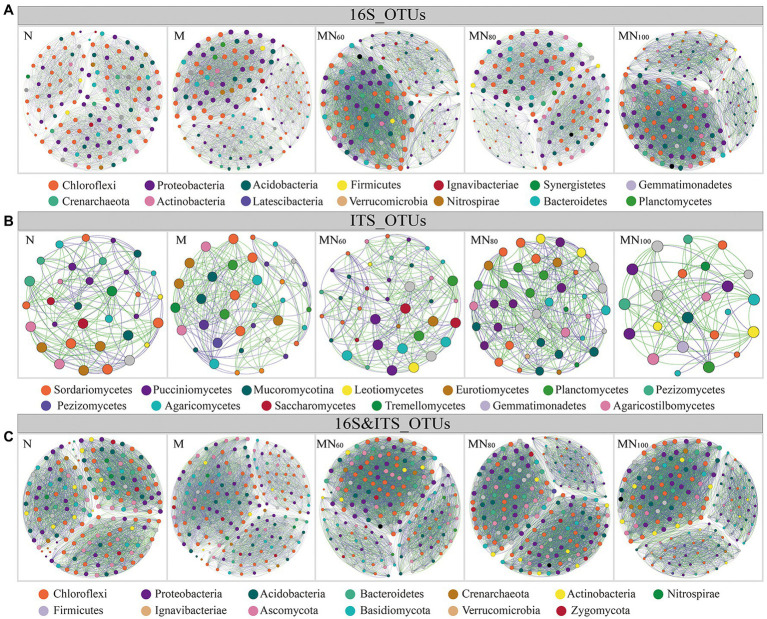
Network visualizes the interactions between soil operational taxonomic units (OTUs) in Karst paddy fields. Positive correlations are displayed in green and negative correlations were displayed in purple. The nodes are colored according to different types of species category. The size of each node is proportional to the betweenness centrality. **A–C** denote bacterial intra-group interactions, fungal intra-group interactions, and bacterial–fungal inter-group interactions, respectively.

Network was used to analyze the dominant bacterial and fungal groups with relative abundance ≥ 0.1% in different fertilization treatments (Figure 4). Among them, the results of the co-occurrence network within bacterial groups showed that the green manure input increased the bacterial interactions compared with N (Figure 4A). However, the number of positively correlated edges decreases in MN_100_ compared with MN_60_ and MN_80_ (Supplementary Table 2). In contrast, the interactions within fungal groups showed the opposite trend to that of bacteria (Figure 4B). According to parameters such as number of nodes, total connected edges and modularity, the change in complexity of bacterial and fungal networks under different fertilization treatments followed the same trend as the change in bacterial diversity. Compared with N treatment, bacterial network complexity was greatest at MN_100_ and second highest at MN_80_, while fungal networks showed the opposite trend (Figure 4). However, for bacterial-fungal interactions, network complexity appeared to be unaffected by microbial abundance.

### The relationships between soil properties, microbial abundance and diversity, and rice yield

#### Relationship between microbial community and environmental properties

**Figure 5:**
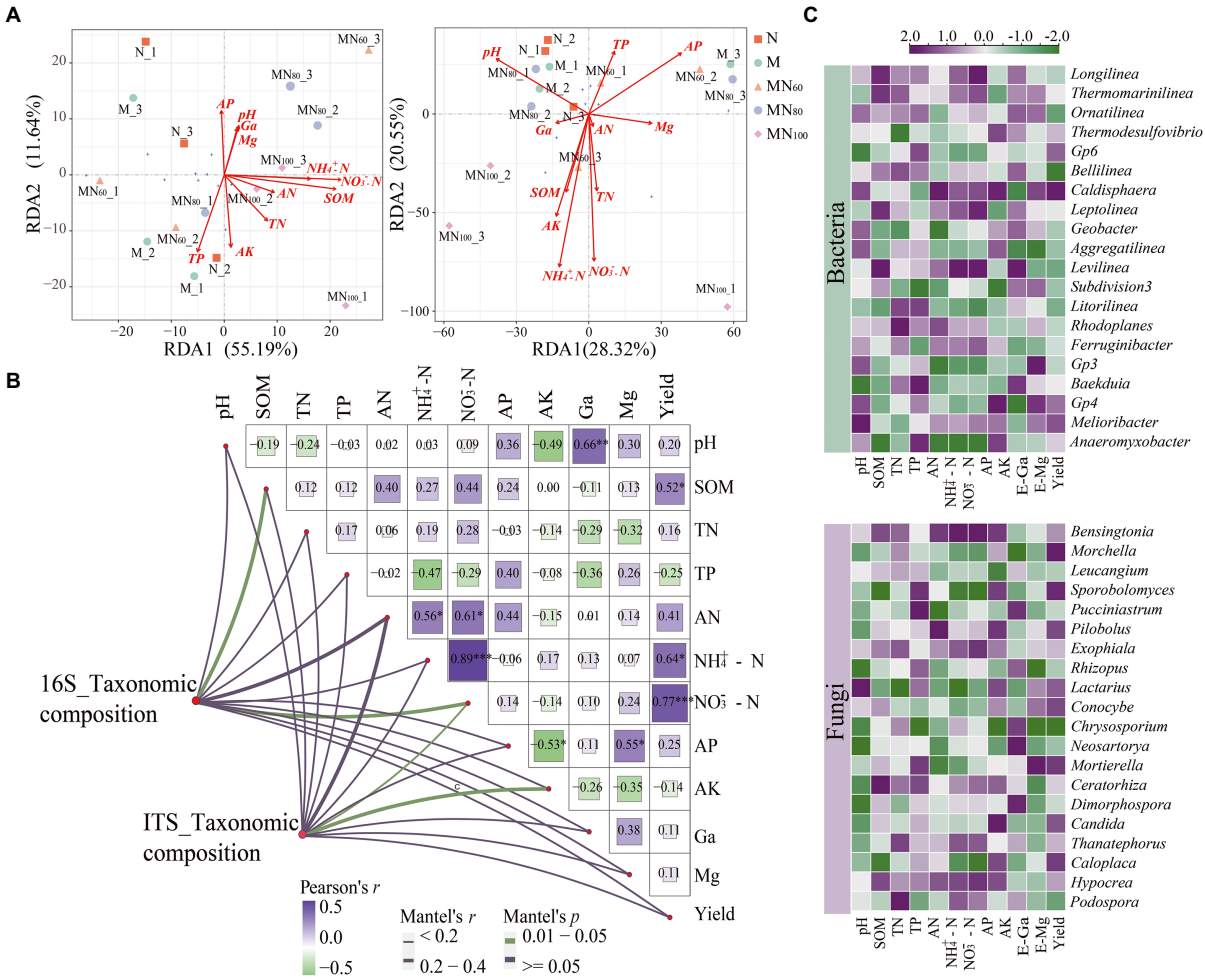
**(A)** RDA analysis of the effect of environmental factors on microbial composition. **(B)** Mantel test above with a Pairwise comparisons of environmental factors are shown, with a color gradient denoting Pearson’s correlation coefficient. Edge width corresponds to the Mantel’s R statistic for the corresponding distance correlations, and edge color denotes the statistical significance based on permutations. **(C)** Heatmap of the correlation between dominant microorganisms (genus level) and environmental factors based on Spearman correlation analysis.

The association between environmental factors and microbial communities was assessed using RDA (Figure 5A). The first two RDA dimensions showed a 67.54% variation in bacterial communities. All environmental factors except AP and TP were positively correlated with RAD1. Among them, SOM (*r* = 0.39, *p* = 0.048) and NO_3_^−^–N (*r* = 0.42, *p* = 0.033) were the environmental factors that significantly affected bacterial communities. And, the first two RDA dimensions explained 48.87% of the variation in bacterial communities, NO_3_^−^–N (*r* = 0.42, *p* = 0.033) were the most important drivers of fungal communities. The Mantel test results showed that SOM (*r* = 0.24, *p* = 0.047), NO_3_^−^–N (*r* = 0.32, *p* < 0.005), NH_4_^+^–N (*r* = 0.17, *p* = 0.024) and AK (*r* = 0.21, *p* = 0.022) were the key factors driving the changes of bacterial communities (Figure 5B).

The results of Spearman-based correlation heat map show that *Bensingtonia*, *Exophiala*, *Thanatephorus*, and *Hypocrea* showed significantly negative correlations with NH_4_^+^–N and NO_3_^−^–N. *Meanwhile*, *Pucciniastrum*, *Rhizopus*, *Chrysosporium*, *Neosartorya* and *Dimorphospora* also showed significantly negative correlations with AK. In contrast, *Leucangium* and *Chrysosporium* were significantly positively correlated with AP.

### Potential factors affecting sustainable development of rice in karst areas

**Figure 6:**
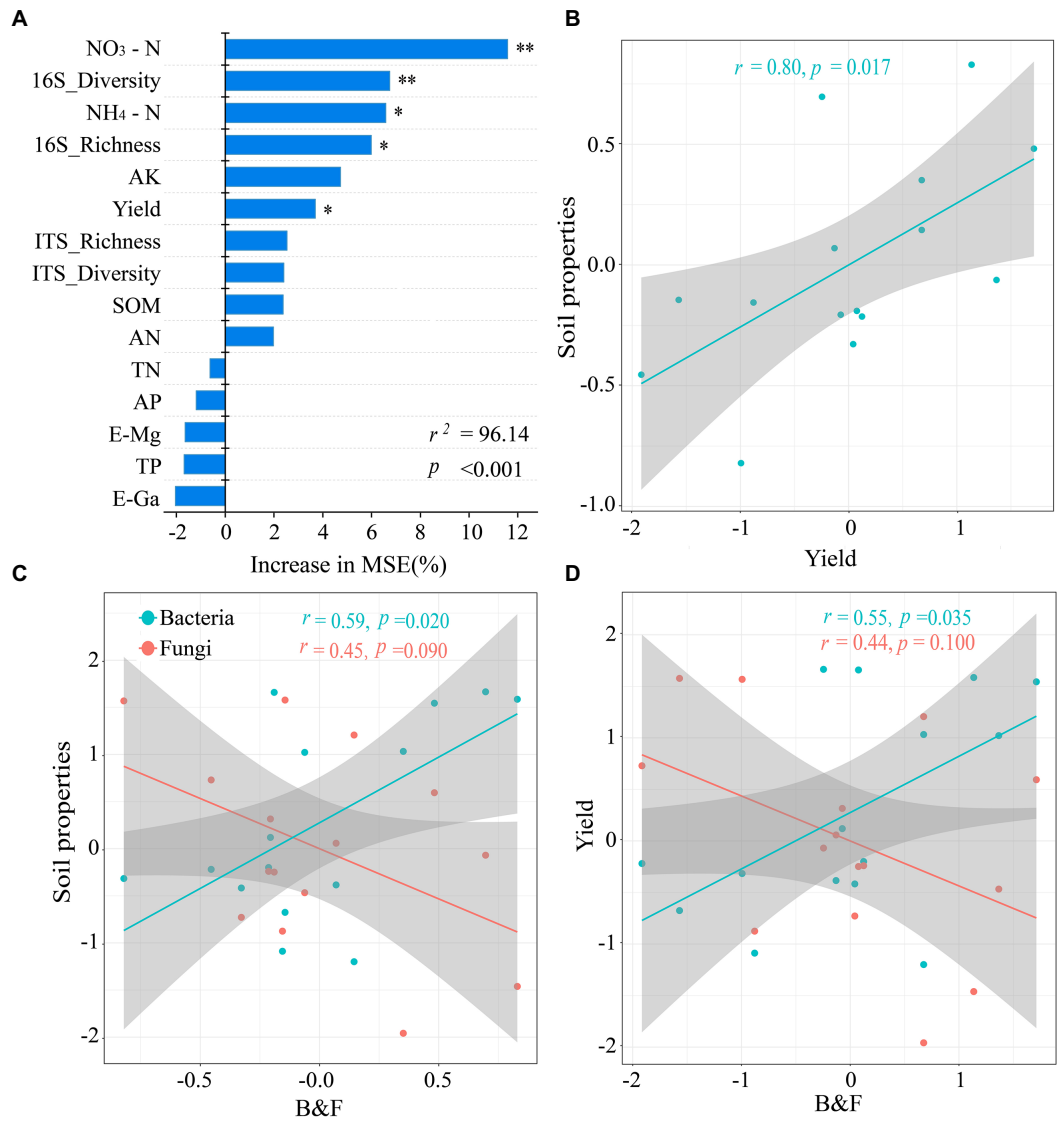
**(A)** Random Forest model showing the importance of predictors under different fertilization treatments. **(B-D)** Regression analysis after standardization (Z-Score) of the data showed a two-by-two linear relationship between soil properties, microorganisms and crop yield. MSE: mean square error. *p < 0.05, **p < 0.01 indicates a significant correlation between the factors.

Random forest modeling was conducted to determine the relative importance of soil environment and microorganisms in predicting soil function and crop yield under different fertilization treatment conditions (Figure 6A). The model explained 96.14% of the ecological variation in soil function. The abundance and diversity of NO_3_^−^–N, NH_4_^+^–N, bacterial communities, and rice yield were predicted with greater importance than other factors. In addition, the regression analysis showed that soil properties had significantly positive linear correlation with rice yield and bacterial community abundance, and had significantly negative linear correlation with fungal community abundance (Figure 6).

## Discussion

The soils in karst areas have slow soil formation rate, thin soil layer and fragile soil environment (Yan et al., 2020). Long-term application of single chemical fertilizer can seriously harm soil ecosystem of rice in karst areas. To solve these problems, we have done corresponding research to mitigate this harm expected to improve rice yield and protect soil ecological balance (Zhong et al., 2021; Pu et al., 2022). Previous studies showed that factors associated with high rice yield include climatic environment, soil nutrients and fertilization measures, as well as the regulation effects of soil microorganisms (Hayat et al., 2010; Romaniuk et al., 2011). In this study, we comprehensively characterized the relationship between soil nutrients, microbial communities and rice yield in karst paddy fields. It was reported that green manure with nitrogen fertilizer increased rice yield (Xie et al., 2016; Gao et al., 2020). Additionally, the use of green manure can effectively reduce the need for nitrogen fertilizer and even increase rice yield (Mohanty et al., 2013; Liu et al., 2021). This result related toproduction of abundant nutrients from green manure decomposition, an exogenous additive that improves the soil environment (Zhang D. et al., 2017) and enhances the activity and mineralization of soil microorganisms (Liao et al., 2015; Hu et al., 2018), which ultimately affects rice yield.

Soil pH, organic matter, nitrogen, phosphorus and potassium influence crop growth, nutrient cycling and microbial processes, and are thus used as indicators of soil fertility (Guo Q. et al., 2020; Chen et al., 2021). As changes in chemical composition and concentration of soil physicochemical factors in most ecosystems are the main drivers of environmental disturbances, it is particularly important to understand the links between changes in microbial communities and the ecological environment (Lozupone et al., 2007; Hall et al., 2018). For instance, although soil pH does not directly affect microbial communities, it may directly or indirectly affect soil nutrient variables, which may change microbial structure and diversity (Lauber et al., 2009; Sagova-Mareckova et al., 2015). On the contrary, green manure inputs increase the amount of soil organic matter, which then decomposes producing many weak acids maintain the balance and stability of soil acidity and alkalinity through acid group dissociation and amino protonation (Zhao et al., 2014). In regard to soil nitrogen, green manure can effectively improve soil nitrogen capacity due to its fixation of atmospheric nitrogen (Gao et al., 2020). In this study, although we found no significant difference in soil nitrogen contents between green manure crop rotation and N fertilizer alone, the rice yield of the green manure group was higher, which may indicate that green manure application improved the absorption and utilization of nitrogen by rice (Nie et al., 2019; Qaswar et al., 2019). We also found no significant difference in rice yield between the high and reduced N (20–40%) treatments, and both were higher than N alone and green manure alone, suggesting that green manure may have a partial substitution effect on N fertilizer (Xie et al., 2016; Qaswar et al., 2019; Zhou et al., 2019). Moreover, soil phosphorus and potassium are important nutrients for crop growth and development. Previous studies reported that green manure rotation reduced soil nutrient absorption and enhanced soil phosphorus and potassium content (Yang et al., 2009). However, this study found that under green manure reversion, nitrogen reduction reduced soil fast-acting potassium content, indicating that rice was more efficient in using fast-acting potassium due to the prolonged flooding of paddy fields (Chen et al., 2021).

Soil microorganisms are the basis of Earth’s biosphere and play an irreplaceable role in nutrient cycling and organic matter decomposition (Balser and Firestone, 2005; Lucas et al., 2007). However, differences in response of different microbial taxa to environmental factors cause different adaptations in environmental gradients (Kivlin et al., 2014). Compared with bacterial communities, fungal communities undergo greater variations (Xu et al., 2019) probably because most fungal taxa in soils have a slower turnover pattern and decompose complex organic matter, making fungi more efficient in their use of organic matter (Waldrop and Firestone, 2006). Dominant taxa of karst paddy soil microorganisms (Figure 5C) grow together, share ecological niches, and play an important role in changing environmental factors such as soil’s physical and chemical properties. For instance, Chloroflexi (bacterial taxa) is relatively abundant in agricultural soils and can ferment sugars and polysaccharide into hydrogen and organic acids, which can promote the degradation of plant residues, fix nitrite and reduce nitrate content (Rao et al., 2022). Similar to Acidobacteria, Proteobacteria play an important role in carbon and nitrogen metabolism and are both highly abundant in agricultural soil environments (Kim et al., 2021). Actinobacteria are widely reported as symbiotic or autotrophic nitrogen-fixing colonies with potassium and phosphate solubilization and produce plant growth agents and biocontrol agents with high functional effects in agricultural production (Boubekri et al., 2022). Bacteroidetes are mainly responsible for nitrification processes, including autotrophic metabolism and subsequent nitrite oxidation (Wolinska et al., 2017). In addition, Ascomycota (fungal taxa) is closely relating to soil nitrogen effectiveness (Liu et al., 2022). Similar results were observed in this study based on the significant and positive correlation (*p* < 0.05) between soil AN and the relative abundance of Ascomycota. Basidiomycota are the group of ecologically strategic fungi with high diversity and high abundance in agricultural soils (Kjoller and Rosendahl, 2014). Zygomycota are the group of fungi employing r-strategy, similar to Ascomycota. They grow fast and effectively decompose unstable carbon, implying high C effectiveness and high relative abundance in soil environments with low C/N in agricultural soil (Liu et al., 2022).

Co-occurrence networks are often used to express the mutual co-occurrence and exclusion of microorganisms, and to identify and categorize key microorganisms that are highly relevant to soil function and crop production (Banerjee et al., 2018; Li et al., 2021) and are used to analyze the interspecific relationships of microbial communities (Barberan et al., 2012). The results showed that the network structure of microbial communities in karst paddy soils differed after reintroducing green manure. For example, 20% N reduction treatment increased the complexity of fungal network compared with N fertilization only, while MN_100_ treatment exhibited lower complexity. This result suggests that changes in microbial diversity are linked to changes in networks, i.e., increased microbial diversity increases the complexity of microbial networks (Guo J. et al., 2020). However, the results of bacterial-fungal interaction network showed that microbial network complexity decreased with full N fertilizer application. This result is in contrast to the bacterial and fungal network results, which may indicate that changes in soil microbial networks are not always associated with changes in microbial diversity, but depend on the interactions between microorganisms (Zhou et al., 2011; Yang et al., 2023). Thus, a multidimensional analysis of the uncertain relationship between diversity and network complexity highlights the importance of studying the relationships within and between microbial groups (Zhou et al., 2010). The significant differences in community composition under different fertilization treatments may explain the changes in abundance of key microorganisms (i.e., *Leptolinea*, *Syntrophorhabdus*, *Geobacter*, and *Dimorphospora*) induced by fertilizer application and alterations in rice yield. In addition, the random forest model and linear regression results showed a significant and negative linear relationship between some soil factors(i.e., NO_3_^−^–N, NH_4_^+^–N, and SOM)and rice yield in karst rice ecosystems. This is important for predicting the influencing factors on rice production.

Compared with fungi, bacteria have higher endogenous growth rates, are more resistant to external environmental disturbances (Loeuille et al., 2017), have relatively rapid nutrient turnover and recovery rates, have relatively rapid nutrient turnover and recovery rates, and have greater ecological niche width to stimulate rapid response (Blagodatskaya and Anderson, 1998). However, the dispersal ability of fungi is limited by the growth of their mycelium, which is affected by several factors, including soil particle size, thus, indicating that fungal community dynamics are often limited by more pronounced environmental factors (Fukami et al., 2010). Previous studies showed that soils with high nitrogen are not conducive to fungal growth (Six et al., 2006; Wu et al., 2014). The green manure species tested in this study was milk vetch. It is a leguminous green manure with low carbon: nitrogen ratio that can fix atmospheric nitrogen and return it to soil through biological nitrogen fixation, releasing about 90% of the nitrogen within 1 month after tilling (Zhu et al., 2014). Therefore, it may explain the apparent effect of green manure application on fungal community. Similarly, we observed that MN_100_ significantly increased B/F ratio compared with N. Additionally, we also observed that soil microbial structures after green manure application tended to be dominated by bacteria (Chen et al., 2019), which was contrary to the previous findings (Wan et al., 2013; Xie et al., 2017). Therefore, we speculate that the results may be related to habitat and climatic conditions in our study area. Therefore, a more in-depth study of these vacancies should also be conducted.

Our multidimensional analysis of environmental conditions, microbial communities and agricultural yield revealed the substitutability of green manure as chemical fertilizers in rice plantations. It could lead to reduced chemical nitrogen fertilizer (20–40% N reduction) and ensure satisfactory crop yield. Consequently, it is beneficial to environment and sustainable agricultural economy.

## Conclusion

For typical karst paddy soils, fertilization practices significantly affect soil microbial community structure and alter microbial intra- and inter-group interactions. Green manure input provided more nutrients for microbial growth and development, which further drove microbial growth and material exchange. In addition, green manure with nitrogen fertilizer increased soil nitrogen, which was more favorable to soil bacterial growth, and maintained stable interactions between bacteria and fungi. Based on these findings, we recommend green manure application with reduced nitrogen fertilizer (20–40% N reduction) in karst areas. Currently, the coupling mechanism between fertilization, microorganisms and soil environment in fragile karst soil ecosystem still needs further in-depth investigations, such as regulation of crop production by affecting certain specific microbial groups, and the relationships between dynamic characteristics of microbial communities and ecological functions during production cycle of rice. In addition, to solve these better-designed studies, combining multiple perspectives are needed in future to systematically elaborate the biogeochemical processes of soil ecology in karst paddy fields for improved sustainable rice production.

## Data availability statement

The datasets presented in this study can be found in online repositories. The names of the repository/repositories and accession number(s) can be found at: https://www.ncbi.nlm.nih.gov/, SRR21891143 SRR21891142 SRR21891136 SRR21891135 SRR21891134 SRR21891133 SRR21891132 SRR21891131 SRR21891130 SRR21891129 SRR21891141 SRR21891140 SRR21891139 SRR21891138 SRR21891137. https://www.ncbi.nlm.nih.gov/, SRR21901609 SRR21901608 SRR21901607 SRR21901606 SRR21901605 SRR21901604 SRR21901603 SRR21901602 SRR21901601 SRR21901600 SRR21901599 SRR21901598 SRR21901597 SRR21901596 SRR21901595.

## Author contributions

JP and ZL conducted the experiments. HT and GZ analyzed the data. CW and WD prepared the figures and tables. ZL and TH designed the project and supervised the experiments. JP, ZL, ZJ, and TH drafted the manuscript. All authors contributed to the article and approved the submitted version.

## Funding

This study was funded by the National Key Research and Development Program of China under Grant (No. 2021YFD1700200), Guangxi Key R&D Program Project under Grant (No. GuiKe AB22080068 and No. Guike AD20297091), National Natural Science Foundation of China (No. 41867008), China Agriculture Research System-Green Manure under Grant (No. CARS-22) and the Research and Development Fund of Guangxi Academy of Agricultural Sciences under Grant (No. 2021YT037 and No. 2022ZX08).

## Conflict of interest

The authors declare that the research was conducted without any commercial or financial relationships that could be construed as a potential conflict of interest.

## Publisher’s note

All claims expressed in this article are solely those of the authors and do not necessarily represent those of their affiliated organizations, or those of the publisher, the editors and the reviewers. Any product that may be evaluated in this article, or claim that may be made by its manufacturer, is not guaranteed or endorsed by the publisher.

## References

[ref1] Balser T. C. Firestone M. K. (2005). Linking microbial community composition and soil processes in a California annual grassland and mixed-conifer forest. Biogeochemistry 73, 395–415. doi: 10.1007/s10533-004-0372-y

[ref2] Banerjee S. Schlaeppi K. van der Heijden M. G. A. (2018). Keystone taxa as drivers of microbiome structure and functioning. Nat. Rev. Microbiol. 16, 567–576. doi: 10.1038/s41579-018-0024-1, PMID: 29789680

[ref3] Bao M. He H. Ma X. Wang C. Qiu W. (2018). Effects of chemical nitrogen fertilizer and green manure on diversity and functions of soil bacteria in wheat Field. Acta Pedol. Sin. 55, 734–743. doi: 10.11766/trxb201710270425

[ref4] Barberan A. Bates S. T. Casamayor E. O. Fierer N. (2012). Using network analysis to explore co-occurrence patterns in soil microbial communities. ISME J. 6, 343–351. doi: 10.1038/ismej.2011.119, PMID: 21900968 PMC3260507

[ref5] Blagodatskaya E. V. Anderson T. H. (1998). Interactive effects of pH and substrate quality on the fungal-to-bacterial ratio and QCO2 of microbial communities in forest soils. Soil Biol. Biochem. 30, 1269–1274. doi: 10.1016/s0038-0717(98)00050-9

[ref6] Boubekri K. Soumare A. Mardad I. Lyamlouli K. Ouhdouch Y. Hafidi M.. (2022). Multifunctional role of Actinobacteria in agricultural production sustainability: a review. Microbiol. Res. 261:127059. doi: 10.1016/j.micres.2022.127059, PMID: 35584559

[ref7] Buyer J. S. Sasser M. (2012). High throughput phospholipid fatty acid analysis of soils. Appl. Soil Ecol. 61, 127–130. doi: 10.1016/j.apsoil.2012.06.005

[ref8] Chen Y. Hu N. Zhang Q. Lou Y. Li Z. Tang Z.. (2019). Impacts of green manure amendment on detritus micro-food web in a double-rice cropping system. Appl. Soil Ecol. 138, 32–36. doi: 10.1016/j.apsoil.2019.02.013

[ref9] Chen J. Huang Y. Tang Y. (2011). Quantifying economically and ecologically optimum nitrogen rates for rice production in South-Eastern China. Agric. Ecosyst. Environ. 142, 195–204. doi: 10.1016/j.agee.2011.05.005

[ref10] Chen X. Liu Q. Zhang G. (2021). Effects of different crop rotation modes on soil fertility and rice yield in Taihu region. Jiangsu J. Agric. Sci. 37, 874–883. doi: 10.3969/j.issn.1000-4440.2021.04.009

[ref11] Chen J. Qin W. Chen X. Cao W. Qian G. Liu J.. (2020). Application of Chinese milk vetch affects rice yield and soil productivity in a subtropical double-rice cropping system. J. Integr. Agric. 19, 2116–2126. doi: 10.1016/s2095-3119(19)62858-3

[ref12] Cock P. J. A. Fields C. J. Goto N. Heuer M. L. Rice P. M. (2010). The sanger FASTQ file format for sequences with quality scores, and the Solexa/Illumina FASTQ variants. Nucleic Acids Res. 38, 1767–1771. doi: 10.1093/nar/gkp1137, PMID: 20015970 PMC2847217

[ref13] Deng Y. Jiang Y. Yang Y. He Z. Luo F. Zhou J. (2012). Molecular ecological network analyses. BMC Bioinform. 13:113. doi: 10.1186/1471-2105-13-113, PMID: 22646978 PMC3428680

[ref14] Fukami T. Dickie I. A. Wilkie J. P. Paulus B. C. Park D. Roberts A.. (2010). Assembly history dictates ecosystem functioning: evidence from wood decomposer communities. Ecol. Lett. 13, 675–684. doi: 10.1111/j.1461-0248.2010.01465.x, PMID: 20412280

[ref15] Gao S. Cao W. Zhou G. Rees R. M. (2021). Bacterial communities in paddy soils changed by milk vetch as green manure: a study conducted across six provinces in South China. Pedosphere 31, 521–530. doi: 10.1016/s1002-0160(21)60002-4

[ref16] Gao X. Wan S. Cao J. Hao Y. Huang F. (2012). Comparative investigation of soil microbial activity in the karst and non-karst areas. Earth Environ. 40, 499–504. doi: 10.14050/j.cnki.1672-9250.2012.04.004

[ref17] Gao S. Zhang R. Cao W. Fan Y. Gao J. Huang J.. (2015). Long-term rice-rice-green manure rotation changing the microbial communities in typical red paddy soil in South China. J. Integr. Agric. 14, 2512–2520. doi: 10.1016/s2095-3119(15)61230-8

[ref18] Gao S. Zhou G. Cao W. (2020). Effects of milk vetch (*Astragalus sinicus*) as winter green manure on rice yield and rate of fertilizer application in rice paddies in South China. J. Plant Nutr. Fertil. 26, 2115–2126. doi: 10.11674/zwyf.20375

[ref19] Guo G. Kong W. Liu J. Zhao J. Du H. Zhang X.. (2015). Diversity and distribution of autotrophic microbial community along environmental gradients in grassland soils on the Tibetan plateau. Appl. Microbiol. Biotechnol. 99, 8765–8776. doi: 10.1007/s00253-015-6723-x, PMID: 26084890

[ref20] Guo Q. Liang G. Zhou W. Chen J. Sun J. Wang X.. (2020). Microbiological mechanism of long-term organic fertilization on improving soil biological properties and double rice yields in red paddy soil. J. Plant Nutr. Fertil. 26, 492–501. doi: 10.11674/zwyf.19450

[ref21] Guo J. Ling N. Chen Z. Xue C. Li L. Liu L.. (2020). Soil fungal assemblage complexity is dependent on soil fertility and dominated by deterministic processes. New Phytol. 226, 232–243. doi: 10.1111/nph.16345, PMID: 31778576

[ref22] Hall E. K. Bernhardt E. S. Bier R. L. Bradford M. A. Boot C. M. Cotner J. B.. (2018). Understanding how microbiomes influence the systems they inhabit. Nat. Microbiol. 3, 977–982. doi: 10.1038/s41564-018-0201-z 30143799

[ref23] Hayat R. Ali S. Amara U. Khalid R. Ahmed I. (2010). Soil beneficial bacteria and their role in plant growth promotion: a review. Ann. Microbiol. 60, 579–598. doi: 10.1007/s13213-010-0117-1

[ref24] Hu L. Su Y. R. He X. (2013). Characteristic of soil aggregate structure in different typical soils in karst region of Northwest Guangxi, China. J. Guangxi Normal Univ. 31, 213–219. doi: 10.16088/j.issn.1001-6600.2013.03.008

[ref25] Hu A. Tang T. Liu Q. (2018). Nitrogen use efficiency in different rice-based rotations in southern China. Nutr. Cycl. Agroecosyst. 112, 75–86. doi: 10.1007/s10705-018-9930-x

[ref26] Jordan F. (2009). Keystone species and food webs. Philos. Trans. R. Soc. B Biol. Sci. 364, 1733–1741. doi: 10.1098/rstb.2008.0335, PMID: 19451124 PMC2685432

[ref27] Kim H. S. Lee S. H. Jo H. Y. Finneran K. T. Kwon M. J. (2021). Diversity and composition of soil Acidobacteria and Proteobacteria communities as a bacterial indicator of past land-use change from forest to farmland. Sci. Total Environ. 797:148944. doi: 10.1016/j.scitotenv.2021.148944. doi: 10.1016/j.scitotenv.2021.148944, PMID: 34298360

[ref28] Kivlin S. N. Winston G. C. Goulden M. L. Treseder K. K. (2014). Environmental filtering affects soil fungal community composition more than dispersal limitation at regional scales. Fungal Ecol. 12, 14–25. doi: 10.1016/j.funeco.2014.04.004

[ref29] Kjoller R. Rosendahl S. (2014). Cultivated and fallow fields harbor distinct communities of Basidiomycota. Fungal Ecol. 9, 43–51. doi: 10.1016/j.funeco.2014.02.005

[ref30] Lauber C. L. Hamady M. Knight R. Fierer N. (2009). Pyrosequencing-based assessment of soil pH as a predictor of soil bacterial community structure at the continental scale. Appl. Environ. Microbiol. 75, 5111–5120. doi: 10.1128/aem.00335-09, PMID: 19502440 PMC2725504

[ref31] Li Q. (2022). Microbial mechanism on distribution, renewal, and maintenance of soil organic carbon pool in karst area. Acta Microbiol Sin. 62, 2188–2197. doi: 10.13343/j.cnki.wsxb.20220010

[ref32] Li Z. He T. Tang H. Wei C. Dong W. (2019). Knowledge mapping analysis of green manure research based on CiteSpace. J. Chin. Agric. Mech. 40, 157–164. doi: 10.13733/j.jcam.issn.2095-5553.2019.07.28

[ref33] Li Q. Song A. Yang H. Mueller W. E. G. (2021). Impact of rocky desertification control on soil bacterial Community in Karst Graben Basin, southwestern China. Front. Microbiol. 12:636405. doi: 10.3389/fmicb.2021.636405, PMID: 33790877 PMC8006366

[ref34] Liao Y. Lu Y. Xie J. Zhou X. Nie J. Tang W.. (2015). Effects of combined application of controlled release nitrogen fertilizer and Chinese milk vetch on yield and nitrogen nutrient uptake of early rice. J. Soil Water Conserv. 29, 190–195+201. doi: 10.13870/j.cnki.stbcxb.2015.03.035

[ref35] Liaw A. Wiener M. (2002). Classification and regression by randomForest. R News. 2, 18–22.

[ref36] Liu T. Wu C. Li H. Ning C. Li Y. Zhang X.. (2022). Soil quality and r–K fungal communities in plantations after conversion from subtropical forest. Catena (Amst.) 219:106584:106584. doi: 10.1016/j.catena.2022.106584

[ref37] Liu X. Zhang Y. Han W. Tang A. Shen J. Cui Z.. (2013). Enhanced nitrogen deposition over China. Nature 494, 459–462. doi: 10.1038/nature11917 23426264

[ref38] Liu C. Zhang C. Li B. Lv Y. Nie L. Zhang L. (2021). Effects of *Astragalus sinicus* combined with chemical fertilizer on nitrogen absorption and uti-lization of rice and nitrogen distribution and residue of *Astragalus sinicus* in rice-soil system. Chin. J. Appl. Ecol. 32, 1791–1798. doi: 10.13287/j.1001-9332.202105.026 34042375

[ref39] Loeuille N. Le Mao T. Barot S. (2017). Effects of plant evolution on nutrient cycling couple aboveground and belowground processes. Theor. Ecol. 10, 117–127. doi: 10.1007/s12080-016-0315-y

[ref40] Lozupone C. A. Hamady M. Kelley S. T. Knight R. (2007). Quantitative and qualitative beta diversity measures lead to different insights into factors that structure microbial communities. Appl. Environ. Microbiol. 73, 1576–1585. doi: 10.1128/aem.01996-06, PMID: 17220268 PMC1828774

[ref41] Lu R. (2000). Analytical Method for Soil and Agro-chemical. Agricultural Science and Technology Press, Beijing, China.

[ref42] Lucas R. W. Casper B. B. Jackson J. K. Balser T. C. (2007). Soil microbial communities and extracellular enzyme activity in the New Jersey pinelands. Soil Biol. Biochem. 39, 2508–2519. doi: 10.1016/j.soilbio.2007.05.008

[ref43] Ma X. Wang T. Shi Z. Chiariello N. R. Docherty K. Field C. B.. (2022). Long-term nitrogen deposition enhances microbial capacities in soil carbon stabilization but reduces network complexity. Microbiome. 10:112. doi: 10.1186/s40168-022-01349-1, PMID: 35902889 PMC9330674

[ref44] Mohanty S. Nayak A. K. Kumar A. Tripathi R. Shahid M. Bhattacharyya P.. (2013). Carbon and nitrogen mineralization kinetics in soil of rice-rice system under long term application of chemical fertilizers and farmyard manure. Eur. J. Soil Biol. 58, 113–121. doi: 10.1016/j.ejsobi.2013.07.004

[ref45] Nie J. Yi L. Xu H. Liu Z. Zeng Z. Dijkstra P.. (2019). Leguminous cover crop *Astragalus sinicus* enhances grain yields and nitrogen use efficiency through increased tillering in an intensive double-cropping rice system in Sothern China. Agronomy-Basel. 9:554. doi: 10.3390/agronomy9090554

[ref46] Pan Y. Tian S. Liu D. Fang Y. Zhu X. Zhang Q.. (2016). Reply to comment on "fossil fuel combustion-related emissions dominate atmospheric ammonia sources during severe haze episodes: evidence from N-15-stable isotope in size-resolved aerosol ammonium". Environ. Sci. Technol. 50, 10767–10768. doi: 10.1021/acs.est.6b04197, PMID: 27630056

[ref47] Pu J. Li Z. Zhong J. Jin Z. Tang H. Wei C.. (2022). Effect of the combination of green manure with nitrogen fertilizer on microbial community in karst paddy soil. Acta Microbiol Sin. 62, 2417–2432. doi: 10.13343/j.cnki.wsxb.20220079

[ref48] Qaswar M. Huang J. Ahmed W. Liu S. Li D. Zhang L.. (2019). Substitution of inorganic nitrogen fertilizer with green manure (GM) increased yield stability by improving C input and nitrogen recovery efficiency in Rice based cropping system. Agronomy-Basel. 9:609. doi: 10.3390/agronomy9100609. doi: 10.3390/agronomy9100609

[ref49] Qiu L. Li D. Zhang J. Zhao B. (2021). Effects of key-stone microbe based on co-occurrence networks on wheat yield in the soils with straw returning. Acta Pedol. Sin., 1, 1–13. doi: 10.11766/trxb202107200372

[ref50] Rao M. P. N. Luo Z. Dong Z. Li Q. Liu B. Guo S.. (2022). Metagenomic analysis further extends the role of Chloroflexi in fundamental biogeochemical cycles. Environ. Res. 209:112888. doi: 10.1016/j.envres.2022.112888 35143804

[ref51] Romaniuk R. Giuffre L. Costantini A. Nannipieri P. (2011). Assessment of soil microbial diversity measurements as indicators of soil functioning in organic and conventional horticulture systems. Ecol. Indic. 11, 1345–1353. doi: 10.1016/j.ecolind.2011.02.008

[ref52] Sagova-Mareckova M. Cermak L. Omelka M. Kyselkova M. Kopecky J. (2015). Bacterial diversity and abundance of a creek valley sites reflected soil pH and season. Open Life Sci. 10, 61–70. doi: 10.1515/biol-2015-0007

[ref53] Six J. Frey S. D. Thiet R. K. Batten K. M. (2006). Bacterial and fungal contributions to carbon sequestration in agroecosystems. Soil Sci. Soc. Am. J. 70, 555–569. doi: 10.2136/sssaj2004.0347

[ref54] Sofo A. Ricciuti P. Fausto C. Mininni A. N. Crecchio C. Scagliola M.. (2019). The metabolic and genetic diversity of soil bacterial communities depends on the soil management system and C/N dynamics: the case of sustainable and conventional olive groves. Appl. Soil Ecol. 137, 21–28. doi: 10.1016/j.apsoil.2018.12.022

[ref55] Sun W. Xiao E. Pu Z. Krumins V. Dong Y. Li B.. (2018). Paddy soil microbial communities driven by environment– and microbe–microbe interactions: a case study of elevation-resolved microbial communities in a rice terrace. Sci. Total Environ. 612, 884–893. doi: 10.1016/j.scitotenv.2017.08.275, PMID: 28886540

[ref56] Sunagawa S. Coelho L. P. Chaffron S. Kultima J. R. Labadie K. Salazar G.. (2015). Structure and function of the global ocean microbiome. Science 348, 1261359. doi: 10.1126/science.1261359 25999513

[ref57] Van Der Heijden M. G. A. Bardgett R. D. van Straalen N. M. (2008). The unseen majority: soil microbes as drivers of plant diversity and productivity in terrestrial ecosystems. Ecol. Lett. 11, 296–310. doi: 10.1111/j.1461-0248.2007.01139.x, PMID: 18047587

[ref58] Waldrop M. P. Firestone M. K. (2006). Response of microbial community composition and function to soil climate change. Microb. Ecol. 52, 716–724. doi: 10.1007/s00248-006-9103-3 17061172

[ref59] Wan S. Tang B. Wang Y. Zhou H. Guo X. (2013). Effect of returning quantity of *Astragalus sinicus* to soil on quantity and activity of microbial in paddy soil. Soil Fert. Sci. China., 4, 39–42. doi: 10.11838/sfsc.20130409

[ref60] Wolinska A. Kuzniar A. Zielenkiewicz U. Izak D. Szafranek-Nakonieczna A. Banach A.. (2017). Bacteroidetes as a sensitive biological indicator of agricultural soil usage revealed by a culture-independent approach. Appl. Soil Ecol. 119, 128–137. doi: 10.1016/j.apsoil.2017.06.009

[ref61] Wu K. Lin X. Lin W. (2014). Advances and perspective in research on plant–soil–microbe interactions mediated by root exudates. Chin. J. Plant Ecol. 38, 298–310. doi: 10.3724/SP.J.1258.2014.00027

[ref62] Xie Z. He Y. Tu S. Xu C. Liu G. Wang H.. (2017). Chinese Milk vetch improves plant growth, development and N-15 recovery in the Rice-based rotation system of South China. Sci. Rep. 7, 3577. doi: 10.1038/s41598-017-03919-y 28620216 PMC5472609

[ref63] Xie Z. Tu S. Shah F. Xu C. Chen J. Han D.. (2016). Substitution of fertilizer-N by green manure improves the sustainability of yield in double-rice cropping system in South China. Field Crop Res. 188, 142–149. doi: 10.1016/j.fcr.2016.01.006

[ref64] Xu Y. Dong S. Li S. Shen H. (2019). Research progress on ecological filtering mechanisms for plant community assembly. Acta Ecol. Sin. 39, 2267–2281. doi: 10.5846/stxb201804260946

[ref65] Yan J. Zhou Q. Jiang W. Chen J. Li Q. Li Z. (2020). Variation of cultivable bacterial community structure and the main influencing factors in karst paddy soil under different fertilization regimes. Microbiol. China. 47, 2833–2847. doi: 10.13344/j.microbiol.china.200672

[ref66] Yang Y. Chai Y. Xie H. Zhang L. Zhang Z. Yang X.. (2023). Responses of soil microbial diversity, network complexity and multifunctionality to three land-use changes. Sci. Total Environ. 859:160255. doi: 10.1016/j.scitotenv.2022.160255, PMID: 36402341

[ref67] Yang L. Li T. Zhou C. (2009). Long-term fertilization effect on fraction and content of phosphorus in vegetable soil in plastic film house. J. Soil Water Conserv. 23, 205–208. doi: 10.13870/j.cnki.stbcxb.2009.05.049

[ref68] Yang X. You L. Hu H. Chen Y. (2022). Conversion of grassland to cropland altered soil nitrogen-related microbial communities at large scales. Sci. Total Environ. 816:151645. doi: 10.1016/j.scitotenv.2021.151645, PMID: 34774635

[ref69] Yang L. Zhou X. Liao Y. Lu Y. Nie J. Cao W. (2019). Co-incorporation of rice straw and green manure benefits rice yield and nutrient uptake. Crop Sci. 59, 749–759. doi: 10.2135/cropsci2018.07.0427

[ref70] Yuan D. (2001). On the karst ecosystem. Acta Geol. Sin. Eng. Edn. 75, 336–338.

[ref71] Yuan M. Guo X. Wu L. Zhang Y. Xiao N. Ning D.. (2021). Climate warming enhances microbial network complexity and stability. Nat. Clim. Chang. 11, 343–348. doi: 10.1038/s41558-021-00989-9

[ref72] Zhang D. Fu B. Hu W. Zhai L. Liu H. Chen A.. (2017). Increasing soil nitrogen fixation capacity and crop yield of rice-rape rotation by straw returning. Trans. Chin. Soc. Agric. Eng. 33, 133–140. doi: 10.11975/j.issn.1002-6819.2017.09.017

[ref73] Zhang X. Gao J. Cao Y. Ma X. He J. (2013). Long-term Rice and green manure rotation alters the endophytic bacterial communities of the Rice root. Microb. Ecol. 66, 917–926. doi: 10.1007/s00248-013-0293-1, PMID: 24046075

[ref74] Zhang J. Nie J. Cao W. Gao Y. Lu Y. Liao Y. (2023). Long-term green manuring to substitute partial chemical fertilizer simultaneously improving crop productivity and soil quality in a double-rice cropping system. Eur. J. Agron. 142:126641. doi: 10.1016/j.eja.2022.126641

[ref75] Zhang X. Zhang R. Gao J. Wang X. Fan F. Ma X.. (2017). Thirty-one years of rice-rice-green manure rotations shape the rhizosphere microbial community and enrich beneficial bacteria. Soil Biol. Biochem. 104, 208–217. doi: 10.1016/j.soilbio.2016.10.023

[ref76] Zhao S. Cao C. Li K. Qiu S. Zhou W. He P. (2014). Effects of long-term straw return on soil fertility, nitrogen pool fractions and crop yields on a fluvo-aquic soil in North China. J. Plant Nutr. Fertil. 20, 1441–1449. doi: 10.11674/zwyf.2014.0614

[ref77] Zhong Y. Hu J. Xia Q. Zhang S. Li X. Pan X.. (2020). Soil microbial mechanisms promoting ultrahigh rice yield. Soil Biol. Biochem. 143:107741. doi: 10.1016/j.soilbio.2020.107741

[ref78] Zhong J. Tang H. Li Z. Dong W. Wei C. Li Q.. (2021). Effects of combining green manure with chemical fertilizer on the bacterial community structure in karst paddy soil. J. Plant Nutr. Fertil. 27, 1746–1756. doi: 10.11674/zwyf.2021246

[ref79] Zhou J. Deng Y. Luo F. He Z. Tu Q. Zhi X. (2010). Functional molecular ecological networks. MBio 1, e00169–10. doi: 10.1128/mBio.00169-10, PMID: 20941329 PMC2953006

[ref80] Zhou J. Deng Y. Luo F. He Z. Yang Y. (2011). Phylogenetic molecular ecological network of soil microbial communities in response to elevated CO2. MBio 2, e00122–11. doi: 10.1128/mBio.00122-11, PMID: 21791581 PMC3143843

[ref81] Zhou X. Liao Y. Lu Y. Rees R. Cao W. Nie J.. (2020). Management of rice straw with relay cropping of Chinese milk vetch improved double -rice cropping system production in southern China. J. Integr. Agric. 19, 2103–2115. doi: 10.1016/S2095-3119(21)63779-6

[ref82] Zhou X. Lu Y. Liao Y. Zhu Q. Cheng H. Nie X.. (2019). Substitution of chemical fertilizer by Chinese milk vetch improves the sustainability of yield and accumulation of soil organic carbon in a double-rice cropping system. J. Integr. Agric. 18, 2381–2392. doi: 10.1016/s2095-3119(18)62096-9

[ref83] Zhu B. Yi L. Hu Y. Zeng Z. Lin C. Tang H.. (2014). Nitrogen release from incorporated N-15-labelled Chinese milk vetch (*Astragalus sinicus* L.) residue and its dynamics in a double rice cropping system. Plant Soil 374, 331–344. doi: 10.1007/s11104-013-1808-8

[ref84] Zou C. Wang Y. Yang J. Tang B. Liu Y. Zhang X. (2013). Effects of combined application of chemical fertilizer and Chinese milk vetch (*Astragalus sinicus*) on the microorganism and nutrients of paddy soil. Soil Fert. Sci. China., 6, 28–31. doi: 10.11838/n20130606

