# Supplementary material for: Response of soil microbial communities and rice yield to nitrogen reduction with green manure application in karst paddy areas
Source: Front Microbiol. 2023 Jan 9;13:1070876. doi: 10.3389/fmicb.2022.1070876 (PMC9869043; doi:10.3389/fmicb.2022.1070876)
Supplement: Supplementary file 1 [file Table_1.docx]

**Supplementary information**

**Response of soil microbial communities and rice yield to nitrogen reduction with green manure application in karst paddy areas**

**TABLE S1** Soil properties under different fertilization treatments

| **Treatment** | **N** | **M** | **MN_60_** | **MN_80_** | **MN_100_** |
| --- | --- | --- | --- | --- | --- |
| pH | 7.10±0.04a | 6.89±0.07b | 7.00±0.02ab | 7.10±0.16a | 6.95±0.01b |
| SOM | 30.90±1.25c | 32.37±0.24abc | 31.26±0.64bc | 33.49±1.77a | 33.05±0.75ab |
| TN | 2.02±0.05a | 2.04±0.04a | 2.07±0.05a | 2.06±0.05a | 2.05±0.08a |
| TP | 0.37±0.06a | 0.38±0.04a | 0.34±0.01a | 0.38±0.04a | 0.34±0.03a |
| AN | 139.76±3.58b | 141.49±3.29ab | 144.16±3.81ab | 147.98±2.42a | 146.12±6.03ab |
| AP | 16.19±0.95a | 15.64±2.77a | 16.67±1.03a | 17.77±1.07a | 15.26±0.85a |
| AK | 160.03±9.68b | 160.75±9.81b | 125.87±2.61c | 122.98±10.59c | 180.51±3.48a |
| E-Ga | 8.84±0.74a | 8.13±0.57a | 8.63±0.18a | 8.81±1.17a | 8.41±0.57a |
| E-Mg | 0.56±0.02a | 0.54±0.08a | 0.57±0.00a | 0.58±0.03a | 0.56±0.01a |

N: 100% nitrogen fertilizer alone; M: green manure alone; MN_60_: green manure couple with 60% nitrogen fertilizer; MN_80_: green manure couple with 80% nitrogen fertilizer; and MN_100_: green manure couple with 100% nitrogen fertilizer. SOM: soil organic matter; TN: soil total nitrogen; TP: soil total phosphorus; AN: available nitrogen; AP: available phosphorus; AK: available potassium; E-Ga: exchangeable calcium; E-Mg: exchangeable magnesium. The value is mean ± standard error, and different letters indicate significant difference between treatments (*p*< 0.05).

**TABLE S2** Microbial co-occurrence network topology index

|  | **Topological index** | **N** | **M** | **MN_60_** | **MN_80_** | **MN_100_** |
| --- | --- | --- | --- | --- | --- | --- |
| Bacteria | Total nodes | 127 | 136 | 126 | 137 | 140 |
|  | Total edges | 2394 | 3238 | 3283 | 3306 | 3737 |
|  | Positive edges | 50.50% | 49.88% | 51.11% | 50.60% | 49.64% |
|  | Modularity index | 0.659 | 0.570 | 0.376 | 0.590 | 0.460 |
| Fungi | Total nodes | 33 | 36 | 40 | 44 | 23 |
|  | Total edges | 169 | 225 | 247 | 311 | 97 |
|  | Positive edges | 59.76% | 62.67% | 55.47% | 53.38% | 77.32% |
|  | Modularity index | 0.636 | 0.486 | 0.665 | 0.629 | 0.453 |
| Bacteria & Fungi | Total nodes | 166 | 177 | 170 | 187 | 171 |
|  | Total edges | 3907 | 5252 | 5202 | 5734 | 4887 |
|  | Positive edges | 51.70% | 49.52% | 50.72% | 49.36% | 49.55% |
|  | Modularity index | 0.666 | 0.556 | 0.476 | 0.600 | 0.504 |
